# ﻿Taxonomic considerations of selected Western Palaearctic Mordellidae Latreille, 1802 (Coleoptera, Tenebrionoidea)

**DOI:** 10.3897/zookeys.1207.119398

**Published:** 2024-07-12

**Authors:** Enrico Ruzzier, Andrea Di Giulio

**Affiliations:** 1 Department of Science, Università Roma Tre, viale G. Marconi 446, 00146 Rome, Italy Università Roma Tre Rome Italy; 2 National Biodiversity Future Center (NBFC), 90133 Palermo, Italy National Biodiversity Future Center (NBFC) Palermo Italy

**Keywords:** Biodiversity, faunistic, Italy, synonymy

## Abstract

The present contribution is devoted to the review of some species of Mordellidae belonging to the Italian fauna described by Mariano Zuccarello Patti and Mario Enrico Franciscolo. New taxonomic combinations are proposed: *Mediimordaargyropleura* (Franciscolo, 1942), **comb. nov.**, *Mordellaquomoi* Franciscolo, 1942, **comb. rev.**, and *Mordellokoilesgrandii* Franciscolo, 1942, **stat. nov.***Mediimordaargyropleura*, *Mordellaquomoi* and *Mordellokoilesgrandii* are considered species that are not part of the Italian fauna. In addition, given the impossibility of identifying the species based on its original description and the destruction of type material, *Mordellaaradasiana* Patti, 1840 is treated as *nomen dubium*.

## ﻿Introduction

Mordellidae Latreille, 1802, also commonly known as tumbling flower beetles, are members of the rather diverse beetle family that comprises more than 2000 extant species worldwide. This group is notoriously taxonomically complicated due to the great uniformity of species and the fact that some characters have not always been consistently and uniquely applied; this is particularly true for the Western Palaearctic, and Europe in particular, where the great richness in sibling and cryptic species has resulted in high taxonomic productivity as well as increasing difficulty in recognizing species (e.g. Emery 1876; Schilsky 1895; Ermisch 1956, 1962, 1963a, 1963b, 1963c, 1965, 1966, 1969a, 1969b, 1969c, 1977). Therefore, reviewing type material and solving nomenclatural issues (e.g. Kaszab 1979; Horák 1990; Leblanc 2007; Selnekovič and Kodada 2019, Selnekovič and Improta 2020), as well as clarifying the distribution of some species (e.g. Batten 1976a, 1976b; Borowiec 1996; Horák et al. 2012; Ruzzier et al. 2017; Selnekovič and Ruzzier 2019) are essential in this context.

The purpose of this contribution is to review some mordellid taxa described by Mariano Zuccarello Patti and Mario Enrico Franciscolo in order to clarify the identity and assess status of these taxa.

## ﻿Material and methods

All the material considered in this contribution belongs to the collections of the Museo Civico di Storia Naturale “G. Doria”, Genoa, Italy (MSNG).

Measurements are abbreviated in the text as follows: EL, elytral length from scutellar apex to elytral apices along suture; EW, maximum elytral width at humeri; HL, head length from anterior clypeal margin to occipital carina along midline; HpygL, maximum hypopygidial length; HW, maximum head width; PL, pronotal length along midline; PW, maximum pronotal width; PygL, maximum pygidial length; TL, sum of head, pronotal, elytral, and pygidial lengths. Wing venation terminology follows that of Lawrence et al. (2021, 2022).

The morphological study was carried out using an Olympus SZX 16 stereomicroscope. Images were taken with a Visionary Digital LK Lab System (Visionary Digital, Palmyra, VA, USA) equipped with a Canon EOS 6D mark II dSLR camera and an MP-E 65mm f/2.8 1–5× lens (Canon, Tokyo, Japan). Stack images were produced using Helicon Focus v. 7.

## ﻿Taxonomic treatment

### ﻿Tribe Mordellini Latreille, 1802

#### ﻿ Mediimorda argyropleura

Taxon classificationAnimaliaColeopteraMordellidae

(Franciscolo, 1942b) comb. nov.

F8366BF3-78FD-5109-8499-9A63757AF082

Mordella argyropleura Franciscolo, 1942b: 18.
Mordella argyropleura : Franciscolo 1949: 52.
Variimorda (Variimorda) argyropleura : Franciscolo 1995: 11; Horák 2020: 86.
Falsopseudotomoxia argyropleura : Ruzzier 2013: 104.

##### Material.

**Figure 1.**
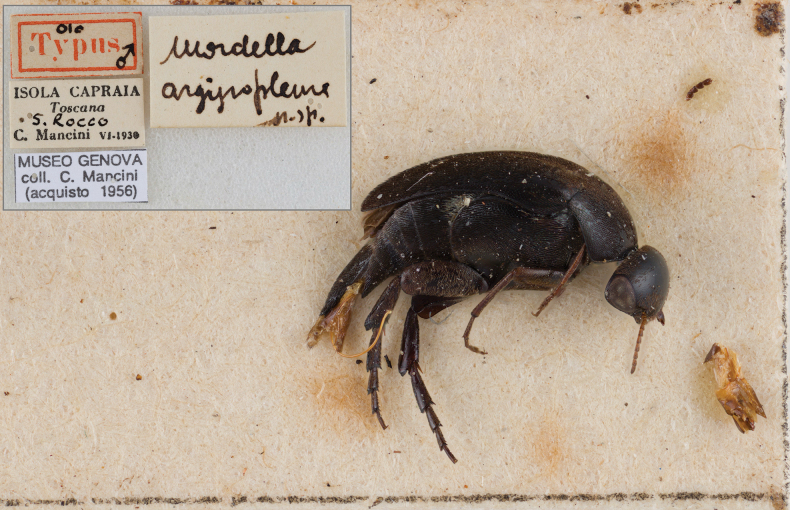
*Mordellaargyropleura* Franciscolo, 1942, holotype.

***Holotype***, male, labeled “Isola di Capraia, Toscana, S. Rocco, C. Mancini, Giglio, VI.1930 // Olo-Typus // *Mordellaargyropleura* n. sp. (handwritten)” (MSNG) (Fig. 1).

Conservation state: state of preservation discreet, left antenna broken, partially missing. The left paramere is damaged and missing the long external branch.

##### Additional material.

12 exx labeled “Somalia IT. Jach Sciumo (Giuba): 1923, S. Patrizi lg. // *Mediimordaargyropleura* E. Ruzzier det. 2024” (MSNG).

##### Comments.

*Mordellaargyropleura* was known to this day exclusively by its type material (Franciscolo 1942b) and the few specimens mentioned in the 1949 contribution (Franciscolo 1949; Ruzzier 2013). The opportunity to review further material in the Genoa Museum’s collection, including the former Franciscolo collection, allowed us to identify additional specimens belonging to this “seemingly elusive” species. Interestingly, all the additional specimens studied originated from Somalia, at that time an Italian colony, and collected by Saverio Patrizi (Latella 2009).

Given this latest finding and given the fact that the type of *M.argyropleura* appears to be prepared on the same kind of paper board as the Somali specimens, we consider the locality of the species described by Franciscolo to be the result of a labeling error, and, therefore, *M.argyropleura* should be excluded from the Italian fauna.

**Figure 2.**
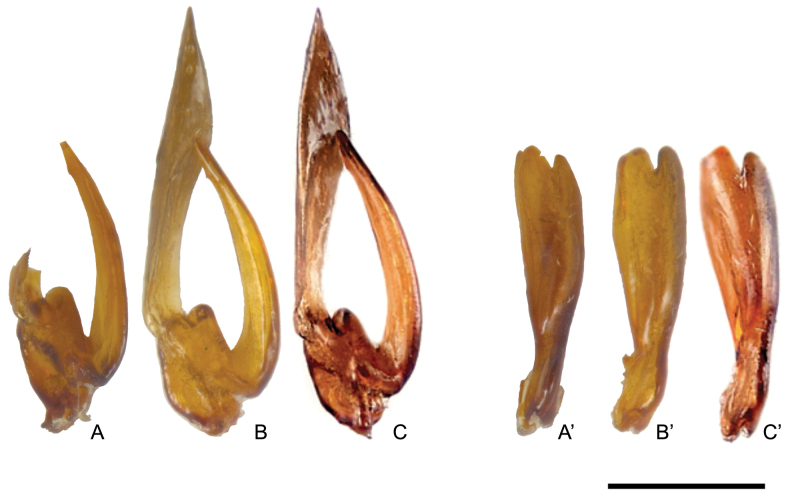
*Mediimordaargyropleura* (Franciscolo, 1942). Holotype of *Mordellaargyropleura* Franciscolo, 1942 **A** right paramere and **A**’ left paramere; male specimen from Jach Sciumo (Giuba, Somalia) **B** right paramere and **B**’ left paramere; male specimen from southern Italy (localities given by Franciscolo 1949, image modified from Ruzzier 2013) **C** right paramere and **C**’ left paramere. Scale bar: 0.2 mm.

Having already had the opportunity to review the type material of *M.argyropleura* (Fig. 1), and after careful consideration based on the comparison of the shape of the paramera (Fig. 2), the general body shape, short antennae, glabrous eyes, presence of a longitudinal dorsal comb on hind tibiae, and small, subtrapezoidal scutellum, we propose *Mediimordaargyropleura* (Franciscolo, 1942b), comb. nov.

The assignment of *Mordellaargyropleura* to *Falsopseudotomoxia* Ermisch, 1954 by Ruzzier (2013) is attributable to the need for a redescription and redefinition of *Mediimorda* Méquignon, 1946 and *Falsopseudotomoxia* Ermisch 1954 as possible synonyms and not vicariant genera in the Western Palaearctic and Afrotropical regions. The re-establishment of the taxonomic combination Variimorda (Variimorda) argyropleura (Franciscolo, 1942) by Horák (2020) is to be regarded as an oversight during the process of updating the Palaearctic catalog.

#### ﻿ Mordella aradasiana

Taxon classificationAnimaliaColeopteraMordellidae

Patti, 1840 nomen dubium

C0147F98-E2AB-5F79-89B8-C62B8878C0CF

Mordella aradasiana Patti, 1840: 292.

##### Comments.

The identity of *M.aradasiana* remains, and will remain, unsolved. The species was described in 1840 by Sicilian amateur naturalist Mariano Zuccarello Patti based on a single specimen he collected in the locality “Praja” (eastern coast of Sicily, between Catania and Agnone Bagni) (Patti 1840). The description, given its year, is rather simple and mostly based on color, and the attribution to Mordellidae and the genus *Mordella* Linnaeus, 1758 (the only one uniformly recognized at the time) by the author, who had very little entomological expertise, was made solely because of the presence of an acuminate pygidium.

Based on the scant information provided (e.g., the size and color pattern), it is impossible to assign the species to any Italian and, more generally, circum-Mediterranean mordellid species; in addition, some of the characters provided in the description, such as the occipital part of the head partially covering the pronotum and the elytra not completely covering the wings underneath, would seem to suggest a taxon belonging to *Macrosiagon* Hentz, 1830 (Ripiphoridae Gemminger, 1870), the female of *Macrosiagonmeridionalis* (Costa, 1859) in particular. Unfortunately, some of the chromatic elements, such as the red spots on the elytra, a character not observed in any Italian *Macrosiagon*, do not allow us to attribute *M.aradasiana* to the latter taxon with certainty.

The quality of the descriptions, as well as the validity of the species described by Patti, have already been widely criticized and in many cases disavowed by several authors (e.g., Ragusa 1883, 1904, 1924; Luigioni 1929), confirming the uncertainty and impossibility of identifying a species often described as “fictional” (Romano 2006).

It is also exceedingly important to note that the type of *M.aradasiana*, like the entire Patti collection inclusive of other taxa he described, was lost or destroyed (Romano 2006).

#### ﻿ Mordella quomoi

Taxon classificationAnimaliaColeopteraMordellidae

Franciscolo, 1942 comb. rev.

AA934B98-811B-51AC-90B8-196F2F2071A9

Mordella quomoi Franciscolo, 1942a: 7.
Mordella cuomoi Franciscolo, 1942b: 22 (subsequent unjustified emendation).
Variimorda (Variimorda) quomoi : Horák 1985: 11; Ruzzier 2013: 106; Horák 2020: 87.
Variimorda (Variimorda) quomoi (Franciscolo, 1951 [sic!]): Franciscolo 1995: 11.

##### Material.

**Figure 3.**
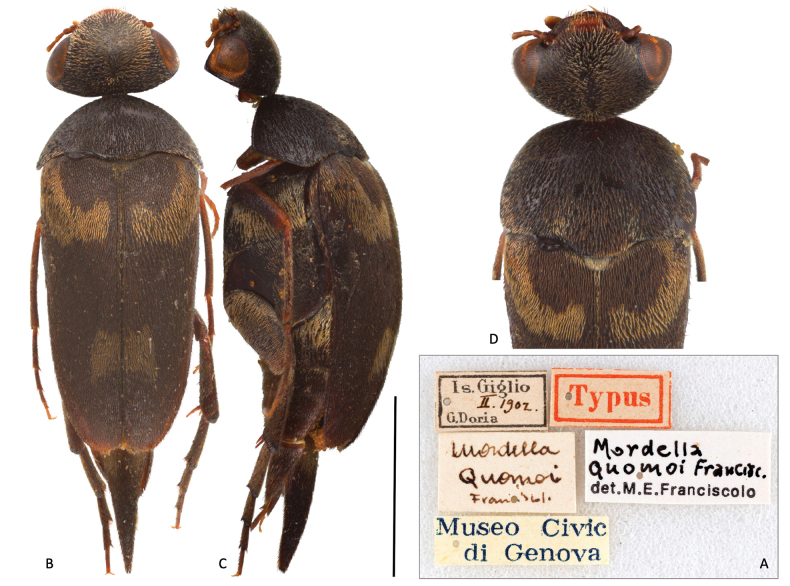
*Mordellaquomoi* Franciscolo, 1942, holotype **A** original labels **B** dorsal habitus **C** lateral left view **D** detail of head and pronotum in dorsal view. Scale bar: 2.0 mm.

***Holotype*** (by monotypy), male, labeled “Is. Giglio, II.1902. G. Doria // Typus // Mordella Quomoi Franciscolo (handwritten)// Mordellaquomoi Francisc. det. M.E. Franciscolo” (MSNG) (Fig. 3A).

Conservation state: modest, antennae and anterior tarsomeres broken, partially missing.

##### Redescription.

***Measurements.***HL: 1.0 mm; HW: 1.5 mm; PL: 1.3 mm; PW: 1.9 mm; EL: 3.3 mm; EW: 1.8 mm; PygL: 1.6 mm; HpygL 0.8 mm; TL: 7.2 mm.

***Color.*** General color of the integument dark brown to black, except for the epistoma, mouthparts, palps, and anterior and middle legs which are orange-amber colored (Fig. 3B, C). Head uniformly covered with golden recumbent setation. Pronotum on disc covered with recumbent, posteriorly oriented, dark-brown setae; margins and the posterior lobe of the pronotum covered with golden setation as on the head. Scutellum bearing pale-yellowish setae. Elytron covered with dark-brown, recumbent setae as on pronotum, except for a band of yellowish setae, which, starting from the scutellum and following the anterior margin of the elytron, delimits the elytral callus and converges toward the elytral suture, forming a semicircular pattern; in addition, the elytron presents a sub-squared patch of yellowish setae at about three-quarters of its length, close to the elytral suture. Meso and metathoracic ventrites, as well as the basal part of all abdominal ventrites, covered with recumbent, yellowish setae.

**Figure 4.**
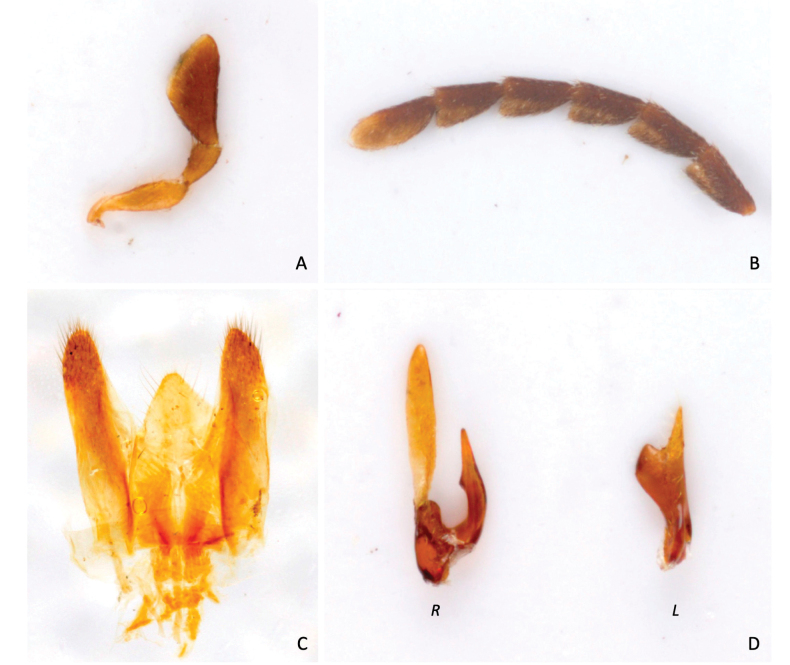
*Mordellaquomoi* Franciscolo, 1942, holotype **A** right maxillary palpus **B** left antennomeres 6–10 **C** 8^th^ sternite **D** paramera, (*R*) right and (*L*) left.

***Head.*** Head transverse in dorsal view (1.6× as wide as long), sub-hemispheric in lateral view with the highest point in correspondence to the occiput (3B); occipital margin almost straight in dorsal view (Fig. 3D); integument nearly smooth but densely covered with setigerous punctures. Eyes large, setose, and finely facetted, almost circular in lateral view, extending anteriorly to the antennal insertion and posteriorly to the occiput. Anterior margin of the epistoma straight; exposed part of exposed part of labrum transverse, gently curved at the anterior margin, setose. Maxillary palpus of the *Mordella*-type (*sensu*Franciscolo 1957) (Fig. 4A); first maxillary palpomere flattened, club-shaped, and about 4× its maximum width; second maxillary palpomere short, subconical, 1.7× as long as its maximum width; apical (third) maxillary palpomere securiform, scalene triangular, 2.1× as long as its maximum width, outer margin of the segment (the longest) feebly sinuate. Antennomeres 1–4 subcylindrical, short; antennomeres 1 and 2 stouter than the following two. Antennomeres 6–10 1.9–2.0× longer than wide (the fifth is missing); apical antennomere asymmetrically ellipsoid, 2.6× as long as wide (Fig. 3B).

***Prothorax.*** Pronotum trapezoid in dorsal view, 1.4× as wide as long, widest slightly before middle, moderately convex in lateral view; pronotal disc microreticulate, densely covered with recumbent and posteriorly oriented setae, punctures larger and more impressed than those on head. Anterior margin of the pronotum gently and gradually curved, and the anterior angles curved downward, resulting in not being visible in dorsal view. Central lobe of the posterior margin of the pronotum markedly protruding posteriad. Anterior and posterior angles of the pronotum obtuse in lateral view; lateral margins of pronotum gently curved in dorsal view, and straight in lateral view. Profemora and protibia gently but markedly curved dorso-ventrally; tarsomeres subcylindrical, truncate at apex.

***Pterothorax*.** Scutellum triangular, with a broadly rounded apex; integument finely punctured and covered with dense and extremely thin setae.

Elytra subconical in dorsal view, external margins feebly but distinctly curving from the elytral humeri towards the apices; elytra apices rounded, not converging at the suture. Elytra strongly and densely punctate; each puncture bears a spiniform seta, especially on the elytral disc.

Metepisterna of the *Mordellistena*-type (*sensu*Franciscolo 1957), triangular, 2.1× as long as their maximum width; sutures between Metepisterna and metaventrite marked and straight. Coxae (coxal plates) semicircular, 2.1× as wide as their maximum length. Metepisterna, metaventrite, and coxae on their dorsal two-thirds densely covered with setigerous punctures like those on pronotum and elytra. Mesofemora laterally compressed; mesotibia cylindrical and feebly curved; mesotibia as long as the mesotarsus. Metafemora straight, laterally compressed; metatibiae conical, transversely truncate at apex; subapical comb present, short and running parallel to the apical margin of the tibia; dorsal side of metatibia bearing a series of sparsely arranged spines that vaguely resemble a longitudinal comb; metatarsomeres devoid of combs or arranged spinulations. Metatibial spurs slightly asymmetrical with the inner 1.1× as long as the outer.

***Abdomen*.** Abdomen conical, tapered in both lateral and dorsal view; abdominal ventrite 1 longer than ventrites 2 to 4, and 1.25× as long as hypopygidium. Pygidium conical in dorsal view, 2.5× longer than wide at the base; in lateral view the pygidium is conical, slightly bending downward towards the base, 2.4× as long as hypopygidium. Sternite 8 strongly produced in the middle of the posterior edge, 1.7× longer than wide at the base, posterior margin bearing long setae (Fig. 3C).

Paramera asymmetric, of the *Mordella*-type (*sensu*Franciscolo 1957) (Fig. 4D). Right paramere U-shaped, dorsal process longer than the ventral, straight, lanceolate and only weakly sclerotized; ventral process short, strongly sclerotized and pointed at apex, presenting a dentiform process on its internal margin. Left paramere stout, claviform, strongly sclerotized, presenting a pointed straight process at the distal apex.

##### Comments.

The species was originally described as *Mordella* based on a single male specimen, but its diagnosis was based exclusively on its external features, color pattern especially (Franciscolo 1942a). Franciscolo named this new taxon after his high-school teacher Maria Enrichetta Cuomo Ulloa (Franciscolo 1942a, 1942b).

In the same year, Franciscolo renamed the taxon *Mordellacuomoi* (Franciscolo 1942b), whose nomenclatural act constitutes in all respects a subsequent unjustified emendation in accordance with Article 33 of the International Code of Zoological Nomenclature (ICZN 1999). The species was then transferred to *Variimorda* Méquignon, 1946 as Variimorda (Variimorda) quomoi by Horák (1985), without taking into account the nomenclature act (despite being incorrect) proposed by Franciscolo 1942; the fact that Horák referred to the holotype as female, when it is clearly male, suggests that the transfer to *Variimorda* was made solely on the basis of the chromatic pattern. Particularly unusual is the assignment by Franciscolo of *M.quomoi* to *Variimorda* in his checklist of the Italian fauna (Franciscolo 1995); the move to another genus is completely unjustified, given the total lack of footnotes or references in the publication, and completely ignoring the taxonomic change proposed by the same author as many as 50 years earlier. Franciscolo then, without referring to the taxonomic change that had already occurred in Horák (1985), proposed “his” combination V. (V.) quomoi, but mistaking its year of description (1951 instead of 1942). The combination Variimorda (Variimorda) quomoi (Franciscolo, 1942) (*sensu*Horák 1985) was then maintained in the few subsequent references mentioning the taxon (Horák 2008; Ruzzier 2013; Horák 2020). Since the species was originally named after a woman, the correct spelling of the specific epithet should have been “*quomoae*” (feminine) instead of “*quomoi*” (masculine); however, we opt to retain the masculine form to maintain consistency with previous literature.

It is important to point out that the species, since its description, has not been found again despite major sampling efforts (E. Ruzzier pers. comm.), and its real identity has remained somewhat uncertain until now.

The possibility of studying the holotype finally allowed us to produce these considerations: 1) the species has to be treated in its original combination since it does not possess those morphological features typical of *Variimorda*, namely antennal dilatation starting from antennomere 5 (antennal dilatation starting from antennomere 4 in *Variimorda*) and left paramere of the *Mordella*-type, short and claviform (knobbed on the inner margin and provided with a well-developed distal membranous process in *Variimorda*); 2) the species does not belong to the Italian fauna, and the locality is most likely the result of a labeling error. This interpretation is supported by the fact that the original specimen label gives February as the month of collection; winter in Italy does not present the climatic conditions to allow the survival of the adults nor favor the presence of flowering plants that could support its survival. In addition, the fact that the species has not been found again in the *locus typicus*, nor in the biogeographically associated and contiguous areas, further supports the idea that the species is not an element of the Italian fauna. Furthermore, its morphological features and general aspect do not resemble any Western Palaearctic member of the tribe Mordellini, with the only exception of a vague affinity with *Mordellariaaurofasciata* (Comolli, 1837). It is currently impossible to determine a plausible origin of this taxon.

### ﻿Tribe Mordellistenini Ermisch, 1941

#### ﻿ Mordellokoiles grandii

Taxon classificationAnimaliaColeopteraMordellidae

Franciscolo, 1942 stat. nov.

66CBDF04-ACC2-5777-8236-2817B951E7C7

Mordellistena (Mordellokoiles) grandii Franciscolo, 1942: 134; Ruzzier 2013: 111.
Mordellokoiles grandii : Franciscolo 1995: 11.
Mordellistena (Mordellokoides) [sic!] grandii: Horák 2008: 96 (incorrect subsequent spelling); Horák 2020: 91 (incorrect subsequent spelling).

##### Material.

**Figure 5.**
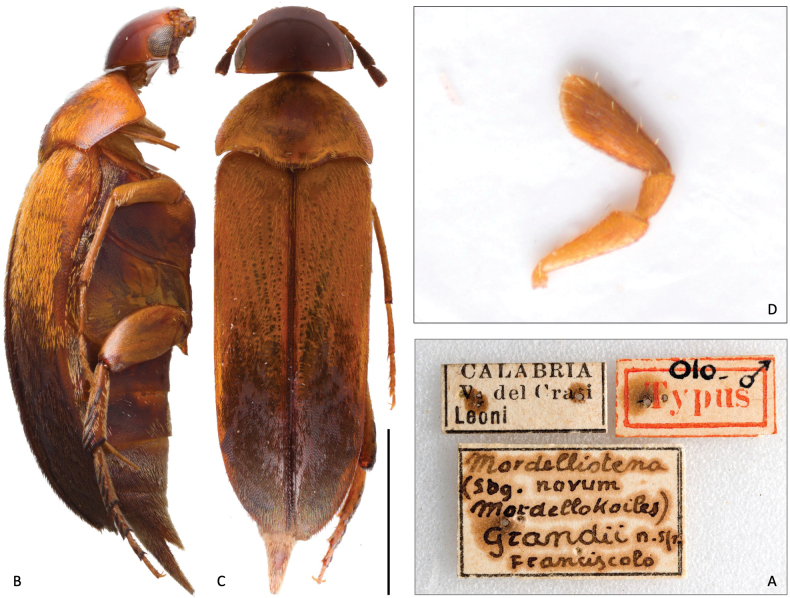
*Mordellokoilesgrandii* Franciscolo, 1942, holotype **A** original labels **B** dorsal habitus **C** lateral left view **D** left maxillary palpus. Scale bar: 2.0 mm.

***Holotype*** (female, not male as stated in the original description) and two paratypes (both females) labeled “Calabria, V. del Crati, Leoni” (MSNG) (Fig. 5A).

Conservation state: the holotype and one paratype are moderately damaged, missing antennomeres, tarsi, and some legs (including posteriors).

##### Redescription.

***Measurements*.** Holotype. HL: 1.2 mm; HW: 1.4 mm; PL: 1.5 mm; PW 1.9 mm; EL: 4.6 mm; EW: 1.9 mm; PygL 1.1 mm; HpygL 0.5 mm; TL: 8.4 mm.

***Color*.** Integument reddish brown to brown; head and ventrites darker (Fig. 5B, C). Mouthparts and the first four basal antennomeres reddish brown; antennomeres from the fifth onwards dark brown (visible only in one paratype). Pronotum presenting a faint longitudinal patch of black, thin setae along the midline; elytra pale reddish brown on their basal half but dark brown on the apical half; dark part of the elytra covered with dark setae.

***Head*.** Head moderately transverse in dorsal view (1.2× wider than long), sub-hemispheric but slightly flattened in lateral view (Fig. 5B), with the highest point just behind the middle of the frons; occipital margin almost straight in dorsal view (Fig. 5C); integument smooth and densely covered with setigerous punctures. Eyes small, setose, and finely facetted, broadly ellipsoidal in lateral view, reaching anteriorly the antennal insertion but not reaching posteriorly the occiput. Anterior margin of the epistoma straight; labrum transverse, gently bisinuate on the anterior margin, setose. Maxillary palpus of the *Mordellistena*-type (*sensu*Franciscolo 1957) (Fig. 5D); first maxillary palpomere flattened, conical and oblong, about 3.8× its maximum width; second maxillary palpomere short, subcylindrical, 1.8× longer than its maximum width; apical (third) maxillary palpomere scalene triangular and oblong, 2.7× longer than its maximum width, outer margin of the segment (the longest) straight.

Antennae filiform (missing in the holotype and one paratype); antennomeres 1–4 cylindrical, with 1 and 2 slightly longer and thicker than 3 and 4; antennomeres 5–10 expanded and only slightly serrated, elongated, 1.7–1.9× longer than wide; apical antennomere asymmetrically ellipsoid, slightly impressed on its inner apical side, 1.9× longer than wide (visible only in one paratype).

***Prothorax*.** Pronotum trapezoidal in dorsal view, 1.3× wider than long, widest on its basal third, slightly convex in lateral view, the convexity increases towards the posterior third of the pronotum; pronotal disc smooth but densely covered with impressed setigerous punctures bearing recumbent and posteriorly oriented setae, punctures larger than those on head. Anterior margin not visible from above since the anterior angles curve downward; in dorsal view, lateral margins of the pronotum roundly and gently converge to the neck. Posterior margin of the pronotum irregular, with the central lobe protruding posteriad. Anterior and posterior angles of the pronotum obtuse in lateral view; lateral margins of pronotum straight in lateral view.

***Pterothorax*.** Scutellum triangular, finely punctured, and covered with dense, thin setae.

Elytra 2.4× longer than wide, parallel in dorsal view (Fig. 5C); external margins markedly curving from the 2/3 of the elytral length towards the apices; elytra apices feebly rounded, almost converging at the suture. Elytra strongly and densely punctate; each puncture bears a spiniform seta, especially on the elytral disc.

**Figure 6.**
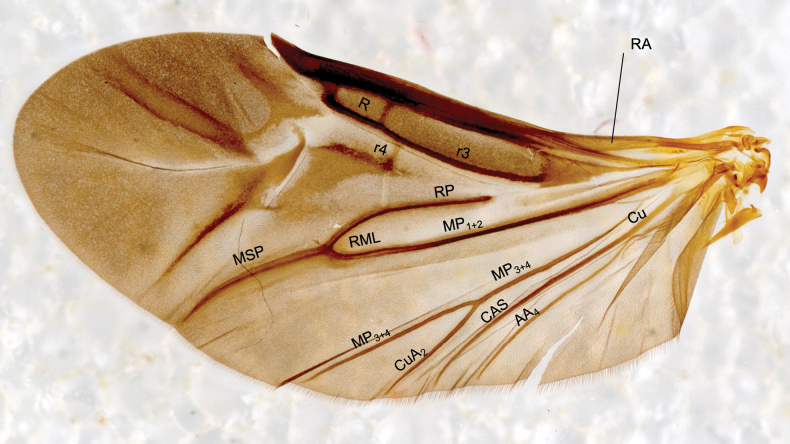
*Mordellokoilesgrandii* Franciscolo, 1942., paratype, right wing, in ventral view.

Hind wing (paratype) of the *Mordellistena*-type (see Fedorenko 2009) (Fig. 6).

Metepisterna of the *Mordellistena*-type (*sensu*Franciscolo 1957), trapezoidal, 2.1× longer than its maximum width; sutures between Metepisterna and metaventrite marked and straight. Coxae (coxal plates) semicircular, and almost as long as wide. Metepisterna, metaventrite, and coxae on their half, densely covered with setigerous punctures like those on pronotum and elytra.

Mesofemora laterally compressed; mesotibia cylindrical and only feebly curved in dorso-lateral view; mesotarsus 1.1× as long as mesotibia. Metafemora straight, laterally compressed; metatibiae conical, transversely truncate at apex; subapical comb present, short and not running parallel to the apical margin of the tibia; dorso-lateral side of metatibia bearing two strongly oblique combs with the proximal longer than the distal. Metatarsomere 1 bearing three oblique, parallel combs, metatarsomere 2 with two, metatarsomere 3 with one comb, and metatarsomere 4 devoid of any. Metatibial spurs asymmetrical with the inner 2.6× as long as the outer.

***Abdomen*.** Abdomen conical, tapered in both lateral and dorsal view; abdominal ventrite 1 almost of the same length as ventrite 2 and 3, ventrite 4 is the shortest. Pygidium conical in dorsal view, 1.8× longer than wide at the base; in lateral view the pygidium is conical, slightly bending downward towards the base, 2.2× as long as hypopygidium.

##### Comments.

*Mordellokoilesgrandii* represents another case of a species described as native to Italy by Franciscolo but never found again since its description. Examination of the type material suggests that this taxon does not belong to the Western Palaearctic fauna and especially poses serious difficulties in framing *Mordellokoiles* with respect to the other genera of Mordellistenini.

As with *Mordellaquomoi*, the description of the new taxon is rather synthetic, not accompanied by adequate iconography, the sex of the holotype is clearly mistaken (male instead of female), and the establishment of the subgenus Mordellokoiles is based on a sole antennal character of rather dubious validity (shape of the last antennomere). Because *M.grandii* possesses the penultimate tarsomere of the anterior and middle tarsi dilated, it cannot be considered in any way as a subgenus of *Mordellistena* Costa, 1854, a genus that possesses cylindrical and apically truncated pro- and mesotarsomeres. Furthermore, this taxon cannot be assigned to any of the Western Palaearctic Mordellistenini with a similar tarsal character, such as *Tolida* Mulsant, 1856, *Dellamora* Normand, 1916, or *Pseudodellamora* Ermisch, 1942, because it differs from these in its general aspect and other characters. However, *Mordellokoiles* is rather similar to some Oriental and Eastern Palaearctic genera, such as *Falsomordellistena* Ermisch, 1941, *Glipostenoda* Ermisch, 1950 or *Pulchrimorda* Ermisch, 1968. However, it cannot be treated as a synonym of any of these because characters required to identify and separate the genera are almost exclusively sexually dimorphic, and it is the male that usually possess the most informative traits. Consequently, any placements of *Mordellokoiles* in any of the already existing genera could be incorrect, as this taxon establishment was based exclusively on females; therefore, we consider it appropriate to elevate *Mordellokoiles* to a genus rank, pending future reassignment if the male is discovered.

## ﻿Discussion

The results presented here demonstrate once again how the examination and redescription of type material is a key step in clarifying the identity and distribution of the Western Palaearctic Mordellidae. The assignment of *Mediimordaargyropleura*, *Mordellaquomoi*, and *Mordellokoilesgrandii* to the Italian fauna by Franciscolo derives most probably from labeling mistakes possibly due to poor management of specimens collected or studied in the early years of his entomological career, as already noted by Magrini and Casale (2015) and Poggi (2023). It is, therefore, possible that the further study of material he has described over the years may result in additional discoveries and corrections in the Italian fauna.

## Supplementary Material

XML Treatment for Mediimorda argyropleura

XML Treatment for Mordella aradasiana

XML Treatment for Mordella quomoi

XML Treatment for Mordellokoiles grandii

## ﻿Additional information

### Author ORCIDs

Enrico Ruzzier https://orcid.org/0000-0003-1020-1247

Andrea Di Giulio https://orcid.org/0000-0003-0508-0751

### Data availability

All of the data that support the findings of this study are available in the main text.
